# A Comparative Study of the Gut Microbiota Associated With Immunoglobulin a Nephropathy and Membranous Nephropathy

**DOI:** 10.3389/fcimb.2020.557368

**Published:** 2020-10-20

**Authors:** Ruijuan Dong, Ming Bai, Jin Zhao, Di Wang, Xiaoxuan Ning, Shiren Sun

**Affiliations:** ^1^Department of Nephrology, Xijing Hospital, The Fourth Military Medical University, Xi'an, China; ^2^Department of Geriatrics, Xijing Hospital, The Fourth Military Medical University, Xi'an, China

**Keywords:** gut microbiota, immunoglobulin a nephropathy, membranous nephropathy, immune dysregulation, gut dysbiosis

## Abstract

The pathogenesis of immunoglobulin A nephropathy (IgAN) and membranous nephropathy (MN) is characterized by immune dysregulation, which is related to gut dysbiosis. The aim of the study was to compare the gut microbiota of patients with IgAN and MN vs. healthy controls. We used 16S rDNA amplicon sequencing to investigate the bacterial communities of 44 patients with kidney biopsy-proven IgAN, 40 patients with kidney biopsy-proven MN, and 30 matched healthy controls (HC). The abundance of *Escherichia-Shigella* and *Defluviitaleaceae_incertae_sedis* were significantly higher in IgAN than in HC, whereas lower abundances were observed for *Roseburia, Lachnospiraceae_*unclassified*, Clostridium_sensu_stricto_1*, and *Fusobacterium*. Furthermore, the abundance of *Escherichia-Shigella, Peptostreptococcaceae_incertae_sedis, Streptococcus*, and *Enterobacteriaceae_*unclassified increased, while that of *Lachnospira, Lachnospiraceae_*unclassified*, Clostridium_sensu_stricto_1*, and *Veillonella* decreased in MN. The abundance of *Megasphaera* and *Bilophila* was higher, whereas that of *Megamonas, Veillonella, Klebsiella*, and *Streptococcus* was lower in patients with IgAN than in those with MN. Analysis of the correlations showed that in the IgAN group, *Prevotella* was positively correlated, while *Klebsiella, Citrobacter*, and *Fusobacterium* were negatively correlated with the level of serum albumin. Positive correlation also existed between *Bilophila* and Crescents in the Oxford classification of IgAN. In the MN group, negative correlation was observed between *Escherichia-Shigella* and proteinuria, *Bacteroides* and *Klebsiella* showed positive correlation with the MN stage. Patients with IgAN and MN exhibited gut microbial signatures distinct from healthy controls. Our study suggests the potential of gut microbiota as specific biomarker and contributor in the pathogenesis of IgAN and MN.

## Introduction

The gut microbiome, residing at intestinal epithelial barriers, is recognized as an important element that contributes to health and disease (Meijers et al., 2019). Dysregulation in the interactions between the gut microbial ecosystem and the adjacent mucosal immune system have been identified in Crohn's disease (CD) and ulcerative colitis (UC) (Xu et al., 2014). Increasing evidence indicates that gut dysbiosis is associated with other immune-mediated diseases, including systemic lupus erythematous (SLE) (Hevia et al., 2014), ankylosing spondylitis (Wen et al., 2017), and rheumatoid arthritis (RA) (Zhang et al., 2015), where abnormal immune response affect sites distant from the gut. However, a mechanistic connection between the gut microbiota and extra-intestinal immune-mediated diseases remains unclear.

IgA nephropathy (IgAN), the most prevalent primary glomerulonephritis (Rodrigues et al., 2017), is characterized by the deposition of IgA1 (particularly, galactose-deficient IgA1) in the glomerular mesangium (Zhang and Zhang, 2018). Galactose-deficient IgA1, supposed to be produced by Peyer patches in the mucosa-associated lymphoid tissue (MALT) (Zhang and Zhang, 2018), is triggered by exposure to commensal or pathogenic bacteria (Magistroni et al., 2015) and is involved in the initial step in the pathogenesis of IgAN. A previous study showed that certain new risk loci for IgA nephropathy are associated with the maintenance of the intestinal epithelial barrier and response to mucosal pathogens (Kiryluk et al., 2014). The complex crosstalk between the gut and the kidney in IgAN has been elucidated and the gut-kidney axis has been supposed (Coppo, 2018). Studies have suggested that gut dysbiosis may contribute to the onset and progression of IgAN; however, no clear causal associations have been demonstrated so far.

Membranous nephropathy (MN), the most common cause of adult-onset nephrotic syndrome worldwide, is an immune-mediated glomerular disease (Couser, 2017). Muscle-type phospholipase A2 receptor (PLA2R) (Beck et al., 2009) and thrombospondintype-1 domain-containing 7A (THSD7A) (Godel et al., 2015) were identified as the target antigens. Autoantibodies to both target antigens are predominantly of the IgG4 subclass (De Vriese et al., 2017). Cationic bovine serum albumin, identified as the main antigen in early-childhoods membranous nephropathy, may derive from microbiota, intestinal cells, and milk formula (Debiec et al., 2011). Clonal expansion of B cells, promoted by MALT (Cesta, 2006), may induce autoantibody production by interacting with genetic polymorphisms (Fresquet et al., 2015). With this background, therefore, we hypothesized that gut dysbiosis is possibly involved in the etiopathogenesis of MN.

In this study, we aimed to investigate the association between the gut microbiota and the pathogenesis of IgAN and MN; toward this, we analyzed the bacterial community composition and diversity in patients and healthy controls using 16S ribosomal DNA Miseq sequencing. We report herein substantial differences in the composition of the gut microbiota between patients and healthy controls. Furthermore, we identify taxonomic biomarkers associated with clinical parameters. This study presents a picture of the microbiota in patients with IgAN and MN and offers specific biomarkers that may potentially contribute to the pathogenesis of IgAN and MN.

## Materials and Methods

### Study Population

We enrolled 44 patients with kidney biopsy-proven IgAN and 40 patients with kidney biopsy-proven MN who did not receive corticosteroids and/or immunosuppressive therapies prior to sampling, along with 30 matched healthy controls. All patients with IgAN and MN were recruited between September 2017 and May 2019 from the inpatient department of the Xijing Hospital. Patients with secondary glomerulonephritis, renovascular disease, gastro-intestinal diseases, pregnancy, and other autoimmune disorders were excluded. Healthy controls were enrolled from physical examination center volunteers. None of the individuals had taken antibiotics or probiotics/prebiotics for at least 3 months prior to sample collection. All individuals confirmed that there was no significant change in meals and other lifestyle-related factors for at least 2 weeks. The demographic data, including gender, age, and body mass index (BMI), clinical features, results of biochemical examination, and biopsy-based pathological manifestations were recorded (Supplementary Table 1).

### Collection of Fecal Samples and DNA Extraction

Fresh fecal samples were collected from the recruited individuals in the morning. After collection, the samples were immediately frozen and stored at −80°C prior to analyses. The fecal sample was added to a 2 ml screwcap vial containing 1 g glass beads (0.1 mm BioSpec Products, Inc., USA) and was suspended in 790 μl sterile lysis buffer (4 M guanidine thiocyanate; 10% N-lauroylsarcosine; 5% N-lauroyl sarcosine-0.1 M phosphate buffer, pH 8.0). The samples were subjected to bead beating for 10 min at maximum speed prior to incubation at 70°C for 1 h. Microbial DNA was extracted using the E.Z.N.A^®^Stool DNA kit (Omega Bio-tek, Inc., GA,USA).

### Polymerase Chain Reaction (PCR), Miseq Sequencing, and Sequence Data Processing

The V3-V4 hypervariable regions of the 16S rDNA were amplified with primers 341F (5′-CCTACGGGNGGCWGCAG-3′) and 805R (5′-GACTACHVGGGTATCTAATCC-3′) using an EasyCycler 96 (PCR) system (Analytik Jena Corp., AG, Germany). PCR was conducted using the following program: 3 min of denaturation at 95°C, 21 cycles of 0.5 min at 94°C (denaturation), 0.5 min for annealing at 58°C, 0.5 min at 72°C, and 5 min at 72°C for a final extension. PCR was performed in a 20-μl reaction system containing 4 μl 5 × Fastpfu buffer, 2 μl 2.5 mM deoxynucleotide triphosphates (dNTPs), 0.8 μl of each primer (5 μM), 0.4 μl TransStart Fastpfu DNA polymerase (TransGen Biotech, Beijing, China), and 10 ng template DNA. The PCR products were detected on a 2% agarose gel, and the band was extracted and purified using the AxyPrepDNA gel (Axygen, CA, USA) and PCR clean-up system. The purified PCR products were mixed. Sequencing was performed on an Illumina MiSeq platform according to the standard protocols of the Shanghai Mobio Biomedical Technology Co. Ltd., China. The raw read data of all the samples have been deposited in the European Bioinformatics Institute European Nucleotide Archive database under the accession number: PRJNA574226.

### Bioinformatics and Statistical Analysis

The raw data were processed using FLASH with the following criteria: (1) longer than 200 bp; (2) <2 mismatches of primers; (3) no ambiguous bases; (4) longer than 10 bp overlap in sequences that were merged according to their sequence.

Operational taxonomic units (OTUs) were clustered with 97% similarity cutoff using UPARSE(version 7.1 http://drive5.com/uparse/) with the following steps: (1) non-repeating sequences were extracted from the optimized sequences; (2) singletons of non-repeating sequences were removed; (3) OTUs were picked at 97% similarity cutoff; (4) OTU composition was created. The Ribosomal Database Project (RDP) Classifier algorithm(http://rdp.cme.msu.edu) was applied to analyze the taxonomy of each 16S rDNA sequence using the SILVA database(http://www.arb-silva.de). After filtering, we generated an average of 49,775 reads for each sample.

Species accumulation curve was plotted to evaluate the sufficiency of sample size and estimate bacterial richness. α-Diversity was determined using OTU analysis and presented using ACE index, chao index, Shannon index, and Simpson index, which were analyzed using the implemented method in the R package “vegan.” The details are showed in the Supplementary Table 2. Bacterial taxonomic comparison at the phylum and genus levels was tested between two groups using Wilcoxon rank sum test, and Kruskal-Wallis rank sum test was used among the three groups, Benjamini-Hochberg control was employed for the false discovery rate. To identify key discriminatory OTUs between the IgAN, MN, and healthy control group, random forest using OTUs as predictors and a mapping file category as class labels were introduced (supervised_learning.py). Heatmap was constructed to select differential OTUs with both the value of Mean_decrease_in_accuracy above 0.001, and the maximum abundance of OTUs above 0.03. β-Diversity was measured using unweighted UniFrac distances. Principal coordinate analysis (PCoA) was performed to display the space between samples. Linear discriminant analysis (LDA) effect size (LEfSe) was used to identify the characteristic microbiota and explain the differences between the patients and healthy controls. Different characterizations were performed with an LDA cut-off of 2.0. For correlation analysis, Spearman's rank test was performed. The two-tailed *t*-test was used to evaluate continuous variables and the Chi-square test was used to compare categorical variables between the two groups. Statistical analyses were performed using SPSS V.20.0 for Windows (SPSS, Chicago, Illinois, USA). *P*-values were considered significant at *P* < 0.05.

## Results

### Clinical Characteristics of All Participants

We recruited 44 patients with IgAN, 40 patients with MN, and 30 healthy controls. All the patients were newly diagnosed with the conditions using kidney biopsy. The clinical characteristics of the participants are shown in Table 1. Age, gender, and BMI were matched between the patients and healthy controls. As expected, serum creatinine level was higher in patients with IgAN than in healthy controls, while the serum levels of total protein and albumin were significantly lower in patients with MN than in healthy controls (Table 1). The median proteinuria of patients with IgAN was 776 mg/24 h, whereas that of patients with MN was 3,623 mg/24 h. About 72.5% of patients with MN presented with positivity of serum anti-PLA2R antibodies. According to the Oxford classification of IgAN, there was modest glomerulonephritis with mild tubulointerstitial injury. Approximately 35(87.5%) patients presented with stage II MN (Table 2).

**Table 1:** Baseline characteristics of study individuals.

**Clinical indexes**	**IgAN (*n* = 44)**	**MN (*n* = 40)**	**HC (*n* = 30)**	***P***
Age, y	34.89 ± 10.74	43.13 ± 13.81	38.60 ± 12.80	0.012
Male sex	20(45.5%)	26(65.0%)	14(46.7%)	0.150
BMI, kg/m^2^	22.95 ± 3.17	23.76 ± 3.15	22.78 ± 2.32	0.310
Hypertension	11(25.0%)	12(30.0%)	-	0.005
Systolic BP, mmHg	126.05 ± 18.31	126.98 ± 18.78	112.33 ± 9.98	0.001
Diastolic BP, mmHg	78.86 ± 15.37	76.38 ± 12.42	71.77 ± 8.21	0.067
WBC, 10^9^/L	6.71 ± 1.71	6.55 ± 1.37	5.59 ± 1.22	0.004
Hb, g/L	136.75 ± 22.77	143.25 ± 20.73	146.53 ± 16.38	0.113
PLT, 10^9^/L	249.02 ± 96.07	250.0 ± 64.60	212.30 ± 57.49	0.079
TP, g/L	69.40 ± 6.68^a^	51.45 ± 8.24^b^	74.45 ± 3.88	<0.001
ALB, g/L	42.10 ± 5.32^c^	26.90 ± 6.77^d^	48.21 ± 3.35	<0.001
S-Cre, umol/L	102.04 ± 32.33^e^	79.73 ± 20.66	83.85 ± 16.99	<0.001
eGFR, mL/min/1.73m^2^	73.30 ± 23.94	98.15 ± 23.24	85.04 ± 18.24	<0.001
Hematuria	44 (100%)	31 (77.5%)	-	-
Nephrotic syndrome	2 (4.5%)	33(82.5%)	-	-
Proteinuria, mg/24 h	776 (384–1,372)	3,623 (2,000–6,493)	-	-
Anti-PLA2R antibody positivity	0 (0%)	29 (72.5%)	-	-

*Continuous data presented as mean ± standard deviation or median [interquartile range]; categorical variables were presented as number (%). One-way ANOVA was used to evaluate continuous variables and Chi-square test was used to compare categorical variables among the three groups. Two-tailed t test was used to evaluate continuous variables and Chi-square test was used to compare categorical variables between the two groups*.

*BMI, body mass index; BP, blood pressure; WBC, white blood cell count; Hb, hemoglobin; Plt, platelet count; TP, total protein; ALB, albumin; S-Cre, serum creatinine; eGFR, glomerular filtration rate; PLA2R, phospholipase A2 receptor*.

*^a, c, e^P values are from t test between IgAN group and HC group, ^b, d^P values are from t test between MN group and HC group. They are statistical different*.

**Table 2:** Pathological features of the patients with IgAN and MN.

**Pathological features**	**IgAN (*N* = 44)**	**Pathological features**	**MN (*N* = 40)**
Oxford classification		Glomerular lesion of MN	
Mesangial hypercellularity (M0/M1)	22/22 (50%/50%)	Stage I, *n* (%)	2(5%)
Segmental glomerulosclerosis (S0/S1)	10/34 (22.7%/77.3%)	Stage II, *n* (%)	35(87.5%)
Endocapillary hypercellularity (E0/E1)	41/3 (93.2%/6.8%)	Stage III, *n* (%)	3(7.5%)
Tubular atrophy/interstitial fibrosis (T0/T1/T2)	34/8/2 (77.3%/18.2%/4.5%)	Stage IV, *n* (%)	0(0%)
Crescents(C0/C1/C2)	37/7/0 (84.1%/15.9%/0%)		

*Categorical variables were presented as number (%)*.

*M, mesangial hypercellularity (M0, <50% of glomeruli show mesangial hypercellularity; M1, >50% of glomeruli show mesangial hypercellularity); S, segmental glomerulosclerosis (S0, absent; S1, present in any glomeruli); E, endocapillary hypercellularity (E0, no endocapillary hypercellularity; E1, any glomeruli show endocapillary hypercellularity); T, tubular atrophy/interstitial fibrosis (T0, 0%-25% of cortical area; T1, 26%-50% of cortical area; T2, >50% of cortical area); C, crescents (C0: absent; C1, 0%-25% of glomeruli; C2, ≥25% of glomeruli)*.

### Richness and Diversity of the Gut Microbiota

**Figure 1:**
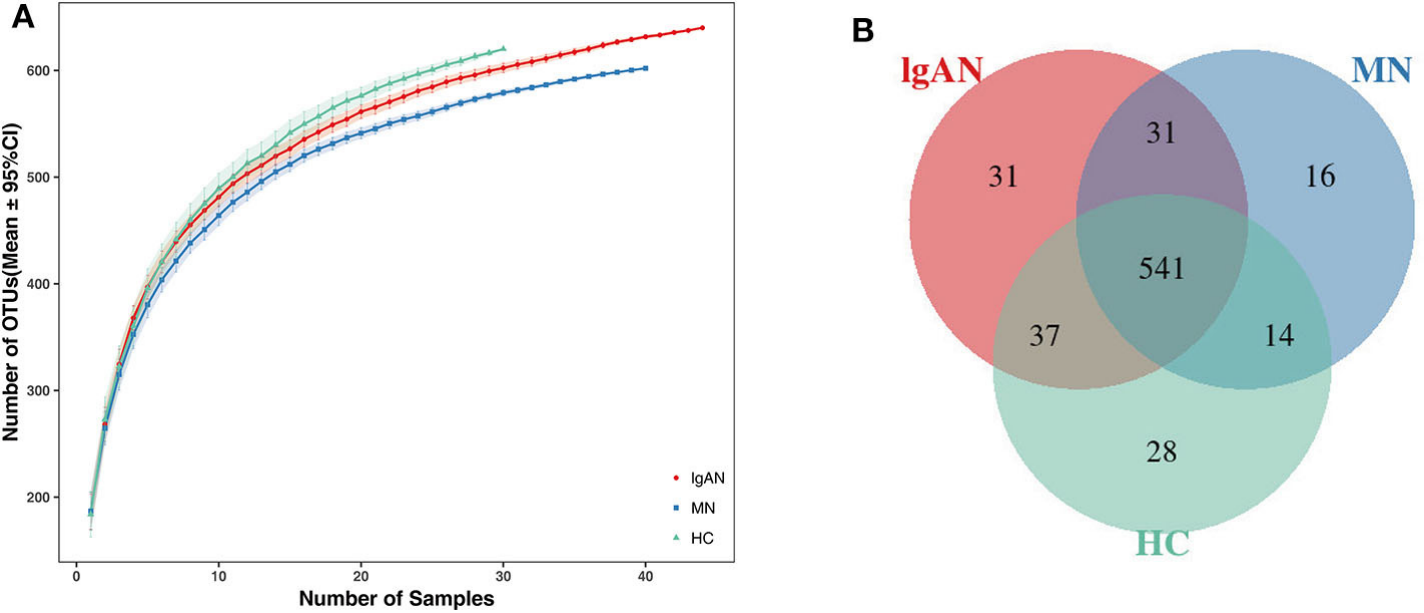
Species accumulation curves and Venn diagram of IgAN, MN and HC groups. **(A)** Species accumulation curves between number of samples and estimated richness. The estimated OTUs richness was close to saturation in each group. **(B)** A Venn diagram displaying the overlaps between groups that 541 of the total 698OTUs were shared among the three groups, while 31 were unique for IgAN, 16 were specific for MN. IgAN, immunoglobulin A nephropathy; MN, membranous nephropathy; HC, healthy controls.

In total, 6,337,125 usable raw reads were obtained from 114 stool samples. After quality filtering and assembly of overlapping paired-end reads, 5,674,350 high-quality reads were generated and 698 operational taxonomic units (OTUs) were obtained based on a 97% homology cutoff. The average number of sequences per sample was 49,775 ± 16,071 (range 25,248–142,345). The values of Good's coverage of all libraries were above 99%. The species accumulation curve showed that the estimated OTU richness already approached saturation at this sequencing depth, suggesting that a vast majority of diversity had been detected (Figure 1A). A Venn diagram showed that 541 of the total 698 OTUs were shared among the three groups, while 31 were unique for IgAN, and 16 were specific for MN (Figure 1B). No significant differences in community richness (estimated by chao and ACE indices) and diversity(measured by Shannon and Simpson indices) were observed between IgAN and the healthy control (Supplementary Table 3, Supplementary Figures 1A–D).Similar trends for community richness and diversity were observed between IgAN and MN, consistent with the trends observed between MN and healthy control (Supplementary Table 3; Supplementary Figures 1A–D).

### Taxonomy-Based Comparisons of Gut Microbiota at the Phylum and Genus Levels

**Figure 2:**
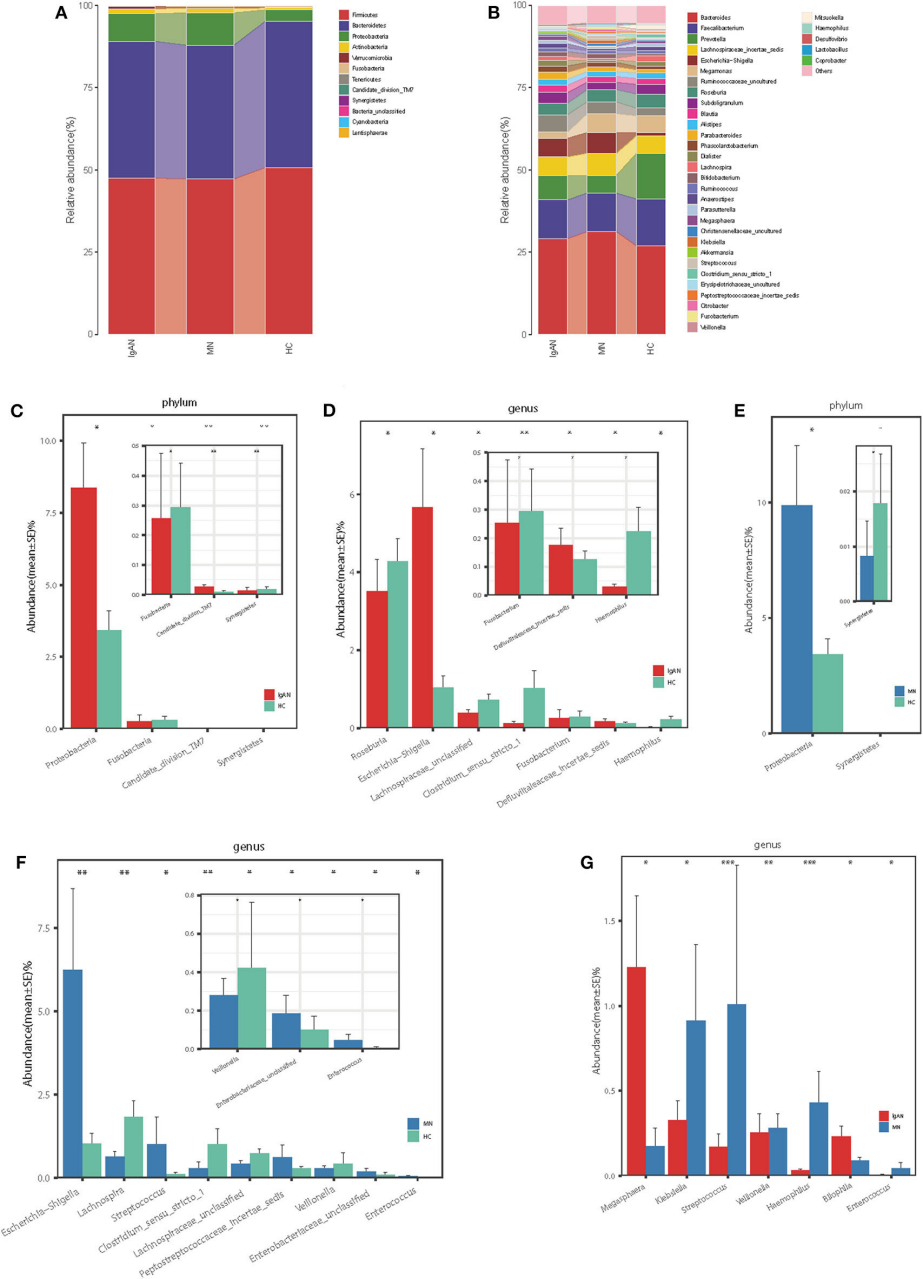
Phylogenetic profiles of gut microbes among patients with IgAN (*n* = 44), patients with MN (*n* = 40) and HC (*n* = 30). Composition of fecal microbiota at the phylum level **(A)** and genus level **(B)** among the three groups. The different microbial community at the phylum level **(C)** and genus level **(D)** in IgAN patients vs. HC. The different microbial community at the phylum level **(E)** and genus level **(F)** in MN patients vs. HC. The different microbial community at the genus level **(G)** in IgAN patients vs. MN patients.*P < 0.05, **P < 0.01. IgAN, immunoglobulin A nedphropathy; MN, membranous nephropathy; HC, healthy controls.

At the phylum level, the gut microbiota of the three groups was dominated by Firmicutes, Bacteroidetes, Proteobacteria, and Actinobacteria, which on average accounted for up to 98% of the relative abundance (Figure 2A). Bacterial genera *Bacteroides, Faecalibacterium, Prevotella*, and *Lachnospiraceae_incertae_sedis*, each accounting for up to 5% of the sequences on average, were the dominant populations (Figure 2B). The fecal microbial composition of all samples at the phylum and genus levels is shown in the Supplementary Figures 2A,B.

### IgA Nephropathy vs. Healthy Controls

Firmicutes was the most predominant phylum, contributing 47.5 and 50.7% of the gut microbiota in patients with IgAN and healthy controls, respectively. Compared to that in the healthy controls, Proteobacteria and Candidate_division_TM7were overrepresented (8.37 vs. 3.41% and 0.026 vs. 0.013%, respectively), while Fusobacteria and Synergistetes were significantly underrepresented in patients with IgAN (0.25 vs. 0.29% and 0.013 vs. 0.018%, respectively, all *P* < 0.05, Figure 2C, Supplementary Table 4). At the genus level, we observed that *Escherichia-Shigella* and *Defluviitaleaceae_incertae_sedis* were enriched in IgAN (5.67 vs. 1.04% and 0.17 vs. 0.12%, respectively), while five genera, namely, *Roseburia, Lachnospiraceae_*unclassified, *Clostridium_sensu_stricto_1, Haemophilus*, and *Fusobacterium* were enriched in the healthy control(3.52 vs. 4.28%, 0.39 vs. 0.72%, 0.12 vs. 1.02%, 0.03 vs. 0.22%, and 0.25 vs. 0.29%, respectively, all *P* < 0.05, Figure 2D, Supplementary Table 5).

### Membranous Nephropathy vs. Healthy Controls

Firmicutes was the dominant phylum in both MN and HC, contributing 47.2 and 50.7% of the gut microbiota, respectively. At the phylum level, the abundance of Proteobacteria increased (9.86 vs. 3.41%), whereas that of Synergistetes decreased in patients with MN compared to that in the healthy controls (0.008 vs. 0.018%, all *P* < 0.05, Figure 2E, Supplementary Table 4). At the genus level, the abundance of five genera, namely, *Escherichia-Shigella, Streptococcus, Enterobacteriaceae*_unclassified, *Peptostreptococcaceae_incertae_sedis*, and *Enterococcus* increased (6.24 vs. 1.04%, 0.61 vs. 0.29%, 1.01 vs. 0.11%, 0.18 vs. 0.10%, and 0.045 vs. 0.007%), whereas the abundance of four genera, namely, *Lachnospira, Lachnospiraceae*_unclassified, *Clostridium_sensu_stricto_1*, and *Veillonella* decreased in patients with MN compared to that in the healthy controls (0.63 vs. 1.83%, 0.43 vs. 0.72%, 0.28 vs. 1.02%, and 0.28 vs. 0.42%, all *P* < 0.05, Figure 2F, Supplementary Table 5).

### IgA Nephropathy vs. Membranous Nephropathy

Next, we investigated how microbial populations vary between disease cohorts, although no significant differences were observed at the phylum level between IgAN and MN. Compared to that in MN, the abundance of genera *Megasphaera* and *Bilophila* increased (1.23 vs. 0.17% and 0.23 vs. 0.09%), whereas those of *Veillonella, Klebsiella, Haemophilus, Enterococcus*, and *Streptococcus* decreased in patients with IgAN (0.25 vs. 0.28%, 0.33 vs. 0.91%, 0.03 vs. 0.43, 0.006 vs. 0.04%, and 0.17 vs. 1.01%, all *P* < 0.05, Figure 2G, Supplementary Table 5).

### Identification of Key OTUs

**Figure 3:**
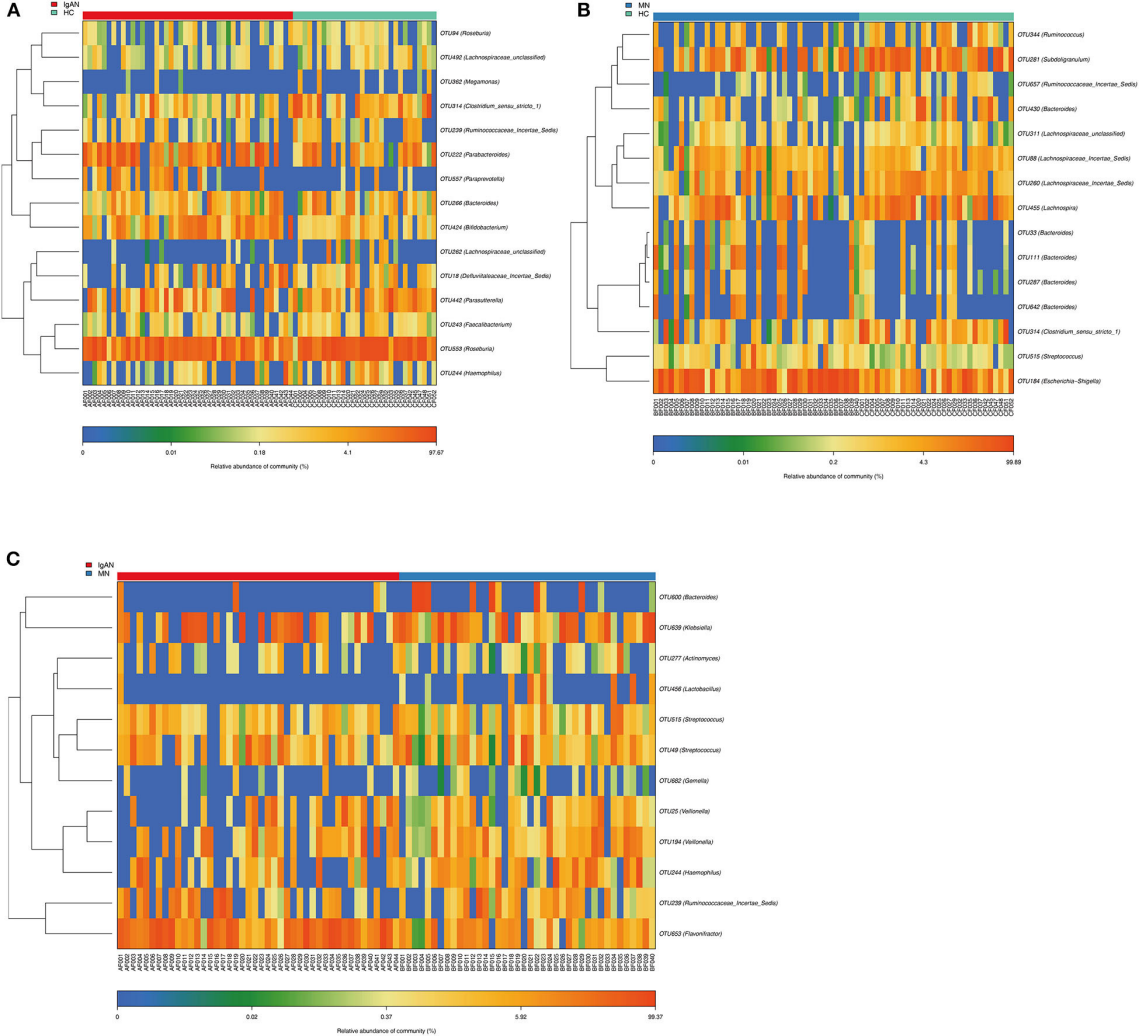
Identification of key Operational Taxonomy Units (OTUs) phylotypes between groups. **(A)** Abundance distribution of the 15 OTUs identified as key phylotypes for IgAN: six OTUs were enriched (orange font), while nine OTUs were decreased (blue font). **(B)** Abundance distribution of the 15 OTUs identified as key phylotypes for MN: seven OTUs were enriched (orange font), while eight OTUs were decreased (blue font). **(C)** Abundance distribution of the 12 OTUs identified as key variables between patients of IgAN and MN: three OTUs were increased in IgAN(orange font), 9 OTUs were increased in MN(orange font).

Compared to that in the healthy control, we identified 15 OTUs discriminatory for IgAN, among which six OTUs assigned to *Bifidobacterium, Paraprevotella, Parabacteroides, Roseburia, Parasutterella*, and *Defluviitaleaceae_incertae_sedis*were enriched, while nine OTUs assigned to *Lachnospiraceae_*unclassified, *Haemophilus, Clostridium_sensu_stricto_1, Bacteroides, Ruminococcaceae_incertae_sedis, Megamonas, Faecalibacterium*, and *Roseburia* were depleted in IgAN. Furthermore, we identified 15 OTUs specific for MN, among which seven OTUs assigned to *Bacteroides, Escherichia-Shigella, Streptococcus*, and *Lachnospiraceae_incertae_sedis* were enriched, while eight OTUs assigned to *Bacteroides, Lachnospiraceae_*unclassified, *Clostridium_sensu_stricto_1, Lachnospira, Lachnospiraceae_incertae_sedis, Ruminococcaceae_incertae_sedis, Subdoligranulum*, and *Ruminococcus* were depleted in MN. Furthermore, we found 12 OTUs as key variables between patients with IgAN and MN, among which three OTUs assigned to *Flavonifractor, Veillonella* and *Ruminococcaceae_incertae_sedis* were enriched in IgAN, while nine OTUs assigned to *Veillonella, Haemophilus, Gemella, Lactobacillus, Bacteroides, Klebsiella, Actinomyces*, and *Streptococcus* were enriched in MN(Figures 3A–C, Supplementary Tables 8–10).

### Differentiation of Patients Based on Gut Microbiota Profiles

**Figure 4:**
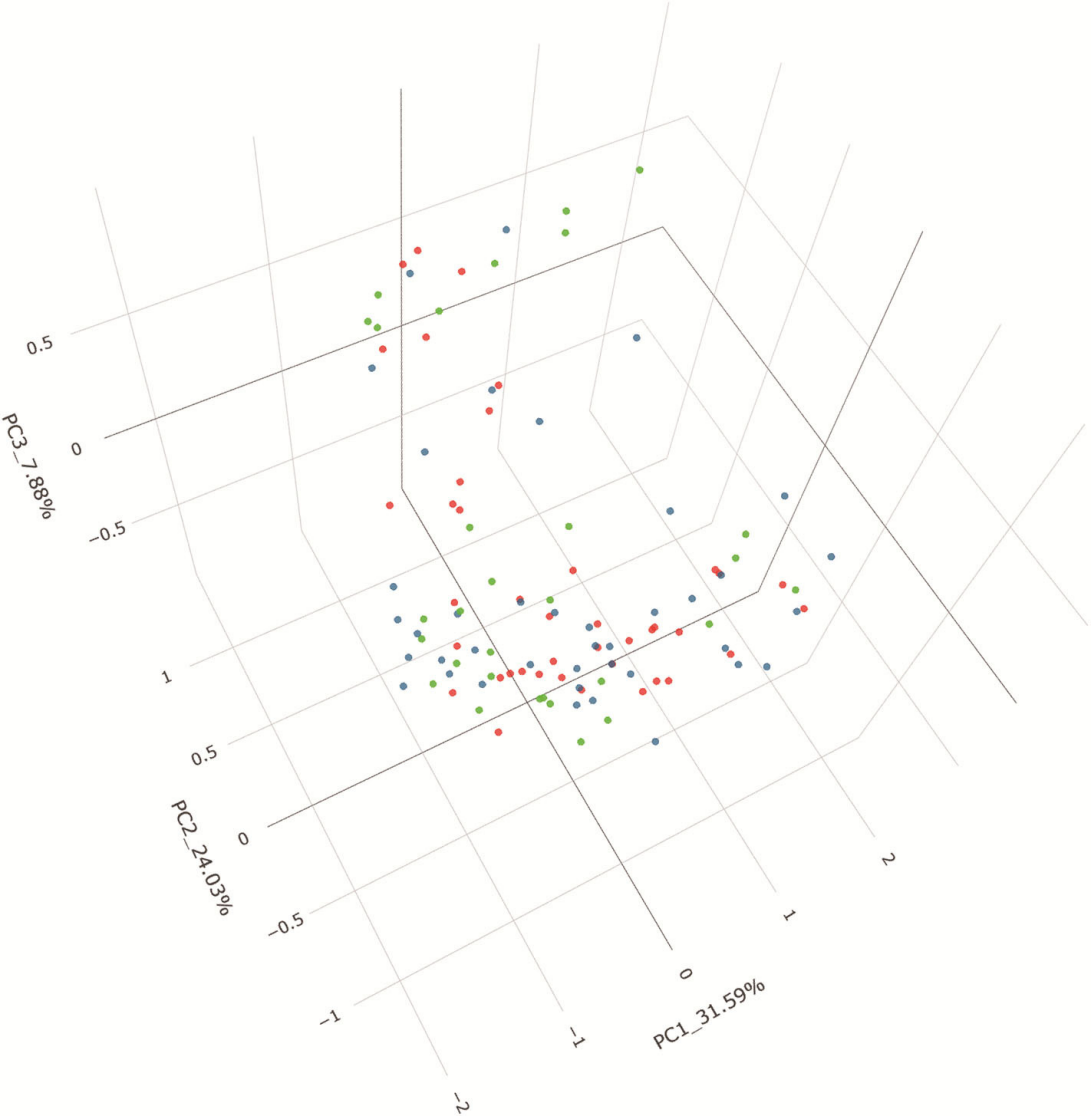
PCoA based on unweighted UniFrac distance. Beta diversity was calculated using unweighted UniFrac by PCoA, indicating a separate distribution of fecal microbial community between IgAN and HC, between MN and HC, a symmetrica distribution between IgAN and MN. PCoA, principal coordinates analysis.

PCoA based on unweighted UniFrac distances revealed that the microbial composition of IgAN deviated from those of the healthy controls (Adonis, *P* = 0.007). The patients with MN and healthy control samples also separated when subjected to PCoA (Adonis, *P* = 0.018). Conversely, a symmetrical distribution was observed between IgAN and MN when subjected to PCoA based on unweighted UniFrac distances (Adonis, *P* = 0.633). All the details are shown in the Figure 4. To identify the specific taxa between groups, we analyzed fecal microbiota using LEfSe. A cladogram presented the gut microbial structures and the major differences in taxa between patients with IgAN and healthy controls (Supplementary Figures 3A,B). We also compared the fecal microbiota to identify the specific taxa between patients with MN and healthy controls (Supplementary Figures 3C,D); results showed gut microbial dysbiosis in patients with IgAN and MN. Further, the cladogram of microbial structure obtained after comparison of the fecal microbiota between disease cohorts showed the maximum differences in taxa (Supplementary Figures 3E,F).

### Spearman Correlation Test in Patients With IgAN and MN

**Figure 5:**
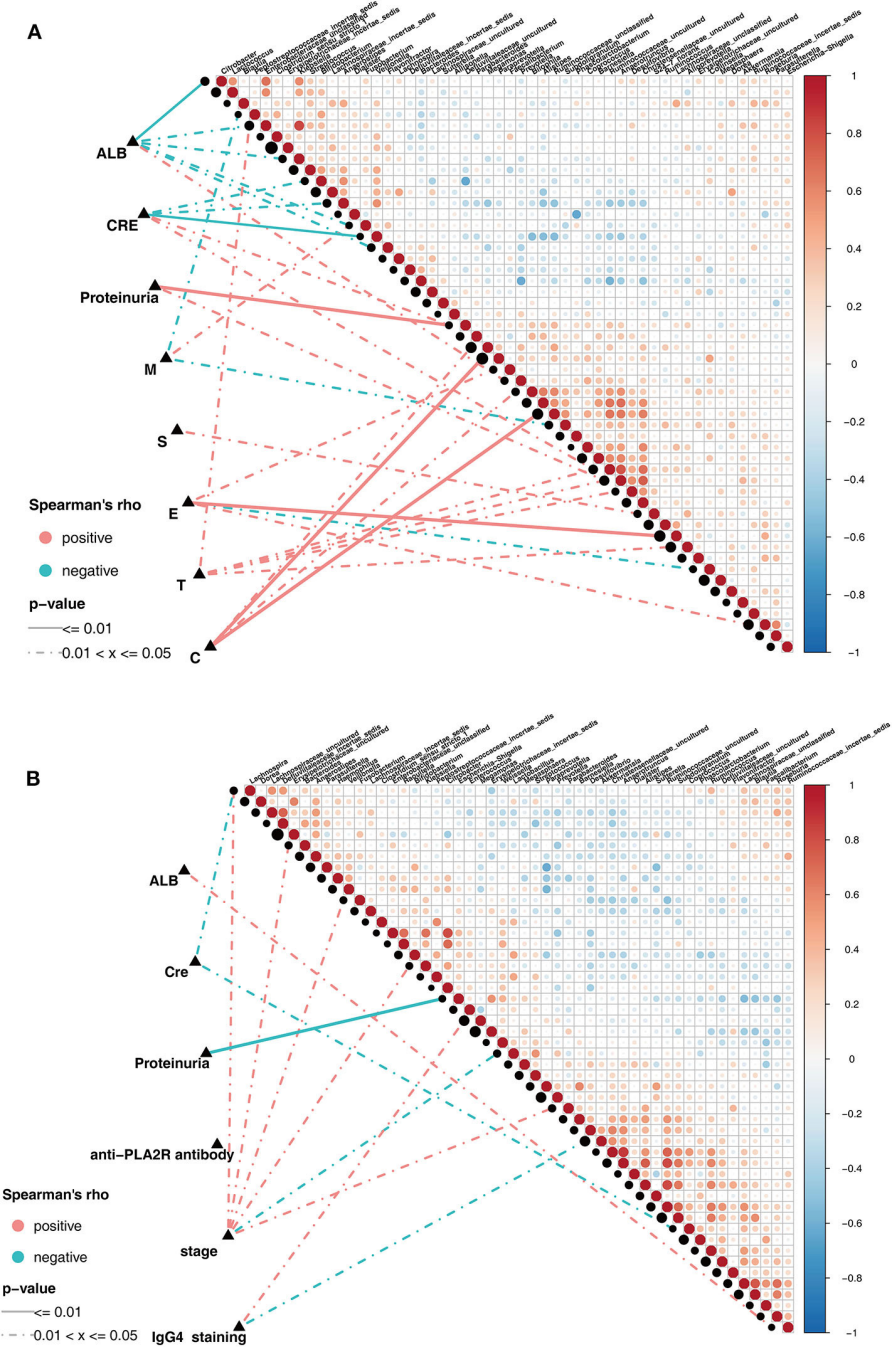
Spearman correlations in patients with IgAN and MN. **(A)** The oblique triangle heatmap indicated ataxon-taxon correlation in IgAN patients, the red circle represented positive correlation, the blue circle represented negative correlation. The correlation network indicated a correlation between faecalmicrobiota and clinical parameters in IgAN patients, the line of solid and dotted indicated strong and weak correlations respectively. **(B)** The oblique triangle heatmap indicated ataxon-taxon correlation in MN patients, the red circle represented positive correlation, the blue circle represented negative correlation. The correlation network indicated a correlation between faecalmicrobiota and clinical parameters in MN patients, the line of solid and dotted indicated strong and weak correlations respectively.

Pairwise comparisons of clinical factors were shown, with a color gradient denoting Spearman's correlation coefficients. The clinical factors were mainly focused on the risk factors for kidney prognosis. In IgAN group, significant positive correlation existed between the genera *Klebsiella* and *Enterobacteriaceae_*unclassified(ρ = 0.82, Figure 5A). In addition, the genus *Prevotella* showed positive correlation, while *Klebsiella, Citrobacter*, and *Fusobacterium* showed negative correlations with serum albumin (ALB) level. Positive correlations existed between *Bilophila* and Crescents in the Oxford classification of IgAN (Figure 5A, Supplementary Table 6). Correspondingly, in the MN group, significant positive correlations existed in the genera: *Alistipes* and *Ruminococcaceae_*uncultured(ρ = 0.82), *Anaerotruncus* and *Christensenellaceae_*uncultured(ρ = 0.87), *Citrobacter* and *Enterobacteriaceae_*unclassified(ρ = 0.81, Figure 5B). Negative correlation existed between *Escherichia-Shigella* and proteinuria, *Bacteroides* and *Klebsiella* showed positive correlation with the MN stage, while *Akkermansia* showed negative correlation with IgG4 deposition in the subepithelia, as observed using immunofluorescence (Figure 5B, Supplementary Table 7).

## Discussion

To our knowledge, our present study represents the characterization of the gut microbiota in patients with IgAN and MN. In our present study, we demonstrated patterns of gut microbiota dysbiosis in two most prevalent glomerulonephritis and identified taxa specific for patients and healthy controls. The results of this study are twofold. First, using differential abundance analyses, we identified that patients with IgAN and MN were characterized by altered composition of the stool microbiota. Second, we showed the dissimilitude of the microbial community between patients with IgAN and MN.

We observed numerous taxonomic differences between disease cohorts and healthy controls. Compared to that in the control, the ethanol-producing genus *Escherichia-Shigella* (Clark, 1989) was enriched, consistent with the previous study (De Angelis et al., 2014), whereas the butyrate-producing genera *Roseburia* and *Faecalibacterium* (Tamanai-Shacoori et al., 2017; Tang et al., 2018)were depleted in patients with IgAN. The opportunistic pathogens *Escherichia-Shigella* occur predominantly in patients with diabetic nephropathy (Tao et al., 2019) and Crohn's disease (Pascal et al., 2017). The increase in *Escherichia-Shigella* population may exacerbate gut leakiness by decreasing butyrate biosynthesis and increasing oxidative stress to penetrate the intestinal epithelial barrier (Croxen et al., 2013).

Compared to that in the healthy controls, the genus *Roseburia* has been reported to be depleted in Crohn's disease (Forbes et al., 2018). Interestingly, patients with Crohn's disease on anti-TNFα antibody therapy showed increase in *Roseburia* abundance (Lewis et al., 2017). Underrepresentation of the genus *Faecalibacterium* has been implicated in several disorders, including inflammatory bowel disease (IBD) (Sokol et al., 2009) and obesity (Turnbaugh et al., 2008). *In vitro*, the culture supernatant of *Faecalibacterium* has been shown to inhibit nuclear factor-κB(NF-κB) activation, which is involved in the pathogenesis of IgAN (Louis and Flint, 2009). As the major source of energy in the intestinal mucosa (Louis and Flint, 2009), butyrate plays an important role in maintaining gut health by exerting anti-inflammatory effects (Tamanai-Shacoori et al., 2017) and affects regulatory T (Treg) cells, which participate in the pathogenesis of IgAN (Ruszkowski et al., 2019). Hence, *Roseburia* and *Faecalibacterium* were reported to be potential markers of gut health (Tamanai-Shacoori et al., 2017; Tang et al., 2018). The reduction in butyrate-producing genera may promote intestinal mucosal destruction (Ren et al., 2019), which plays a critical step in the pathogenesis of IgAN (Kiryluk et al., 2014). Increasing evidence from human studies suggests that *Roseburia* and *Faecalibacterium* are potential probiotic candidates for the treatment of chronic gut inflammation (Sanders et al., 2019).

Interestingly, *Bifidobacterium* abundance was higher in patients with IgAN than in healthy controls. As probiotic microorganisms, members of the genus *Bifidobacterium* are beneficial for health as they affect immune regulation (Round and Mazmanian, 2009), inhibit pathogens (Serafini et al., 2013), and degrade diet-derived carbohydrates (Pokusaeva et al., 2011). Out of more than 50 species assigned to *Bifidobacterium*, only 10 are found in humans. However, a previous study demonstrated the invasive potential of *Bifidobacterium* in immune compromised host (Esaiassen et al., 2017). Another study identified higher abundance of *Bifidobacterium* in patients with ulcerative colitis (Forbes et al., 2018) than in healthy controls. Thus, we propose that some species may be disease-specific and further investigation regarding their effect on gut homeostasis is required.

Investigation of the relationships between clinical parameters and microbial taxa in the IgAN group revealed that *Prevotella* was associated with higher level of ALB, while *Klebsiella, Citrobacter*, and *Fusobacterium* were associated with lower level of ALB. *Prevotella* has previously been reported to be associated with improved glucose metabolism and insulin sensitivity (De Vadder et al., 2016), while *Klebsiella* correlated with the invasion of epithelial cells (Tang et al., 2018).

Compared to that in the healthy controls, the abundance of *Escherichia-Shigella, Bacteroides, Actinomyces*, and *Streptococcus* increased, while those of *Lachnospira* and *Roseburia* decreased in patients with MN. *Escherichia-Shigella* and *Bacteroides* have been reported to produce lipopolysaccharide (LPS) (Wexler, 2007), which initiates various pathophysiological cascades (Darnaud et al., 2013). High levels of LPS activate the NF-κB pathway and lead to the production of pro-inflammatory cytokines(TNF-α, IL-6, and IL-1) (Darnaud et al., 2013). In agreement with this observation, higher abundance of *Escherichia-Shigella* and *Bacteroides* increases the circulating levels of pro-inflammatory cytokines such as TNF-α and IL-6, and genetic polymorphisms in these cytokines are associated with the onset/occurrence of MN (Thibaudin et al., 2007; Chen et al., 2010). *Actinomyces* has been identified to be abundant in patients with rheumatoid arthritis (Thota et al., 2011) and ulcerative colitis (Forbes et al., 2016). Members of the genus *Streptococcus* has recently been associated with numerous immune-mediated inflammatory diseases (Chen et al., 2016; Lewis et al., 2017). Furthermore, the presence of *Streptococcus* may be a predictive marker for the future recurrence of Crohn's disease (Pascal et al., 2017). Butyrate-producing *Lachnospira* and *Roseburia* are potential markers of health. *Akkermansia* displayed negative correlations with IgG4 deposition in the subepithelia. *Akkermansia muciniphila*, which produces the protective mucous lining of the intestine (Berry et al., 2013), is associated with health and the absence of autoimmune diseases (Routy et al., 2018).

Genetic susceptibilities and immune dysregulation are believed to be involved in the pathogenesis of IgAN and MN. Numerous environmental factors are known to affect the gut microbial community. Thus, it is not surprising that the abundance of certain taxa differs in the gut microbiota of patients and healthy controls. It is noteworthy that compared to that in healthy controls, taxa such as *Ruminococcaceae_incertae_sedis* were more abundant in IgAN and less abundant in MN. *Ruminococcaceae* was enriched in patients with hepatic encephalopathy (Bajaj, 2014), although evidence linking *Ruminococcaceae_incertae_sedis* to the disease is lacking. Therefore, it has been hypothesized that the varying abundance of certain taxa may influence autoimmune response and distant organs. Further studies are required to confirm the possible roles these microorganisms in the etiology of glomerulopathy. We identified certain genera (and OTUs) to be uniquely represented in patients compared to in healthy controls. Furthermore, we also identified certain taxa that were significantly different between patients with IgAN and MN. Considering the different pathological patterns of glomerulopathy, it is not surprising that we detected these differences.

Compared to that in the healthy controls, we observed alterations in the gut microbial community in the treatment-naïve IgAN and MN patients, which indicated that certain bacterial candidates may be implicated in disease pathogenesis and can be used as specific biomarkers in the patient cohort.

Despite the promising results, our study has certain limitations. First, we tested a relatively small sample size at the moderate pathological stage; a larger cohort that includes patients at different pathological stages will be necessary to comprehensively study the role of the gut microbiota and further validate these findings. Similarly, sufficient sample size will be required for comparisons across different disease states (active and inactive) and treatments. Data regarding the role of an active immune response and immunomodulating therapies on the gut microbiota are also limited. Second, although age, gender, and BMI were matched in our study, certain confounding effects such as dietary factors, should also be considered. However, the participants in our study were from northwest of China, and the lifestyle factors were similar. Third, we analyzed fecal microbiota, which cannot fully reflect the profiles of mucosal microbiota.

The results of our study demonstrated that alteration of gut microbiota is associated with developments of IgAN and MN, evidenced by the changes in various taxonomic levels. The specific microbes may be potential diagnostic biomarkers and therapeutic targets for IgAN and MN. Potential therapeutic strategies for IgAN and MN that target the gut microbiota by fecal microbiota transplantation are already being investigated.

## Conclusion

In summary, this study presents a comprehensive analysis of the gut microbiota composition in patients with IgAN and MN. We showed that the composition of the gut microbiota differs significantly in patients with IgAN and MN compared to that in the healthy controls. Further investigations are warranted to establish the causality in disease pathogenesis and diagnostic potential.

## Data Availability Statement

The datasets presented in this study can be found in online repositories. The names of the repository/repositories and accession number(s) can be found at: https://www.ebi.ac.uk/ena, PRJNA574226.

## Ethics Statement

The studies involving human participants were reviewed and approved by Ethics Committee of Xijing Hospital of The Fourth Military Medical University. The patients/participants provided their written informed consent to participate in this study.

## Author's Note

This manuscript has been released as a pre-print at (Research square) (Shiren et al., 2020).

## Author Contributions

SS and MB designed, supervised the project, and revised the manuscript for important content. RD and JZ collected samples, performed bioinformatics and statistical analysis, and interpreted data. DW performed pathological diagnosis. RD contributed to data collection and drafted the manuscript. All author contributed important content, accepts personal accountability for the author's own contributions, and agrees to ensure that questions pertaining to the accuracy or integrity of any portion of the work are appropriately investigated and resolved.

## Conflict of Interest

The authors declare that the research was conducted in the absence of any commercial or financial relationships that could be construed as a potential conflict of interest.

## Abbreviations

BMI: body mass index
BP: blood pressure
WBC: white blood cell count
Hb: hemoglobin
Plt: platelet count
TP: total protein
ALB: albumin
S-Cre: serum creatinine
eGFR: glomerular filtration rate
PLA2R: phospholipase A2 receptor.
