# Supplementary material for: A Comparative Study of the Gut Microbiota Associated With Immunoglobulin a Nephropathy and Membranous Nephropathy
Source: Front Cell Infect Microbiol. 2020 Oct 20;10:557368. doi: 10.3389/fcimb.2020.557368 (PMC7606180; doi:10.3389/fcimb.2020.557368)
Supplement: Supplementary file 8 [file Table_8.DOCX]

**Table S8 The comparison of the average percent of the identity OTUs between HC and IgAN**

| ID | HC | lgAN |
| --- | --- | --- |
| OTU553 (Roseburia) | 0.500622902 | 0.335659097 |
| OTU222 (Parabacteroides) | 0.084010423 | 0.231764486 |
| OTU442 (Parasutterella) | 0.10944061 | 0.134731393 |
| OTU424 (Bifidobacterium) | 0.017027522 | 0.114148313 |
| OTU314 (Clostridium_sensu_stricto_1) | 0.136728296 | 0.029816295 |
| OTU266 (Bacteroides) | 0.043467181 | 0.041472755 |
| OTU18 (Defluviitaleaceae_Incertae_Sedis) | 0.024737456 | 0.037270952 |
| OTU244 (Haemophilus) | 0.02961413 | 0.021343069 |
| OTU557 (Paraprevotella) | 0.003945225 | 0.03631281 |
| OTU239 (Ruminococcaceae_Incertae_Sedis) | 0.014859393 | 0.006033695 |
| OTU262 (Lachnospiraceae_unclassified) | 0.015561666 | 3.91E-04 |
| OTU243 (Faecalibacterium) | 0.007719605 | 0.004583398 |
| OTU492 (Lachnospiraceae_unclassified) | 0.005291407 | 0.003275973 |
| OTU94 (Roseburia) | 0.002812813 | 0.002924438 |
| OTU362 (Megamonas) | 0.004161371 | 2.73E-04 |
