# Supplementary material for: A Comparative Study of the Gut Microbiota Associated With Immunoglobulin a Nephropathy and Membranous Nephropathy
Source: Front Cell Infect Microbiol. 2020 Oct 20;10:557368. doi: 10.3389/fcimb.2020.557368 (PMC7606180; doi:10.3389/fcimb.2020.557368)
Supplement: Supplementary file 9 [file Table_9.DOCX]

**Table S9 The comparison of the average percent of the identity OTUs between HC and MN**

| ID | HC | MN |
| --- | --- | --- |
| OTU184 (Escherichia-Shigella) | 0.171481724 | 0.449565807 |
| OTU281 (Subdoligranulum) | 0.269779328 | 0.12499014 |
| OTU455 (Lachnospira) | 0.176102371 | 0.094256998 |
| OTU314 (Clostridium_sensu_stricto_1) | 0.138565012 | 0.040641061 |
| OTU260 (Lachnospiraceae_Incertae_Sedis) | 0.063318515 | 0.043869112 |
| OTU88 (Lachnospiraceae_Incertae_Sedis) | 0.032811417 | 0.051014356 |
| OTU430 (Bacteroides) | 0.086253538 | 0.008577264 |
| OTU111 (Bacteroides) | 0.00570245 | 0.066673742 |
| OTU642 (Bacteroides) | 0.002203527 | 0.039795388 |
| OTU515 (Streptococcus) | 0.004264395 | 0.026712611 |
| OTU344 (Ruminococcus) | 0.027445545 | 0.007934272 |
| OTU33 (Bacteroides) | 0.003265699 | 0.019561318 |
| OTU287 (Bacteroides) | 0.002798941 | 0.019018162 |
| OTU311 (Lachnospiraceae_unclassified) | 0.008422275 | 0.006009708 |
| OTU657 (Ruminococcaceae_Incertae_Sedis) | 0.007585264 | 0.001380061 |
