# Supplementary material for: A Comparative Study of the Gut Microbiota Associated With Immunoglobulin a Nephropathy and Membranous Nephropathy
Source: Front Cell Infect Microbiol. 2020 Oct 20;10:557368. doi: 10.3389/fcimb.2020.557368 (PMC7606180; doi:10.3389/fcimb.2020.557368)
Supplement: Supplementary file 10 [file Table_10.DOCX]

**Table S10 The comparison of the average percent of the identity OTUs between IgAN and MN**

| ID | lgAN | MN |
| --- | --- | --- |
| OTU639 (Klebsiella) | 0.223577454 | 0.244094041 |
| OTU653 (Flavonifractor) | 0.329364774 | 0.08416442 |
| OTU49 (Streptococcus) | 0.088329414 | 0.099422442 |
| OTU194 (Veillonella) | 0.088253585 | 0.08854706 |
| OTU244 (Haemophilus) | 0.053448141 | 0.114664136 |
| OTU239 (Ruminococcaceae_Incertae_Sedis) | 0.089409777 | 0.062680124 |
| OTU600 (Bacteroides) | 0.013070176 | 0.14646511 |
| OTU25 (Veillonella) | 0.062095121 | 0.045029044 |
| OTU515 (Streptococcus) | 0.03834074 | 0.049745024 |
| OTU456 (Lactobacillus) | 0.001370332 | 0.039052193 |
| OTU277 (Actinomyces) | 0.01007155 | 0.020995039 |
| OTU682 (Gemella) | 0.002668935 | 0.005141368 |
