# Supplementary material for: A Comparative Study of the Gut Microbiota Associated With Immunoglobulin a Nephropathy and Membranous Nephropathy
Source: Front Cell Infect Microbiol. 2020 Oct 20;10:557368. doi: 10.3389/fcimb.2020.557368 (PMC7606180; doi:10.3389/fcimb.2020.557368)
Supplement: Supplementary file 11 [file Data_Sheet_1.PDF]

Supplemental Fig. 1 Community richness and diversity among the three groups.

Supplemental Fig. 2 Composition of faecal microbiota among IgAN(n=44), MN(n=40) and HC(n=30).

Supplemental Fig. 3 The specific characterization of gut microbiota to identify taxa was analyzed by linear discriminant analysis (LDA) effect size(LEfSe) method between IgAN (n=44) and HC(n=30), between MN(n=40) and HC, between IgAN and MN.

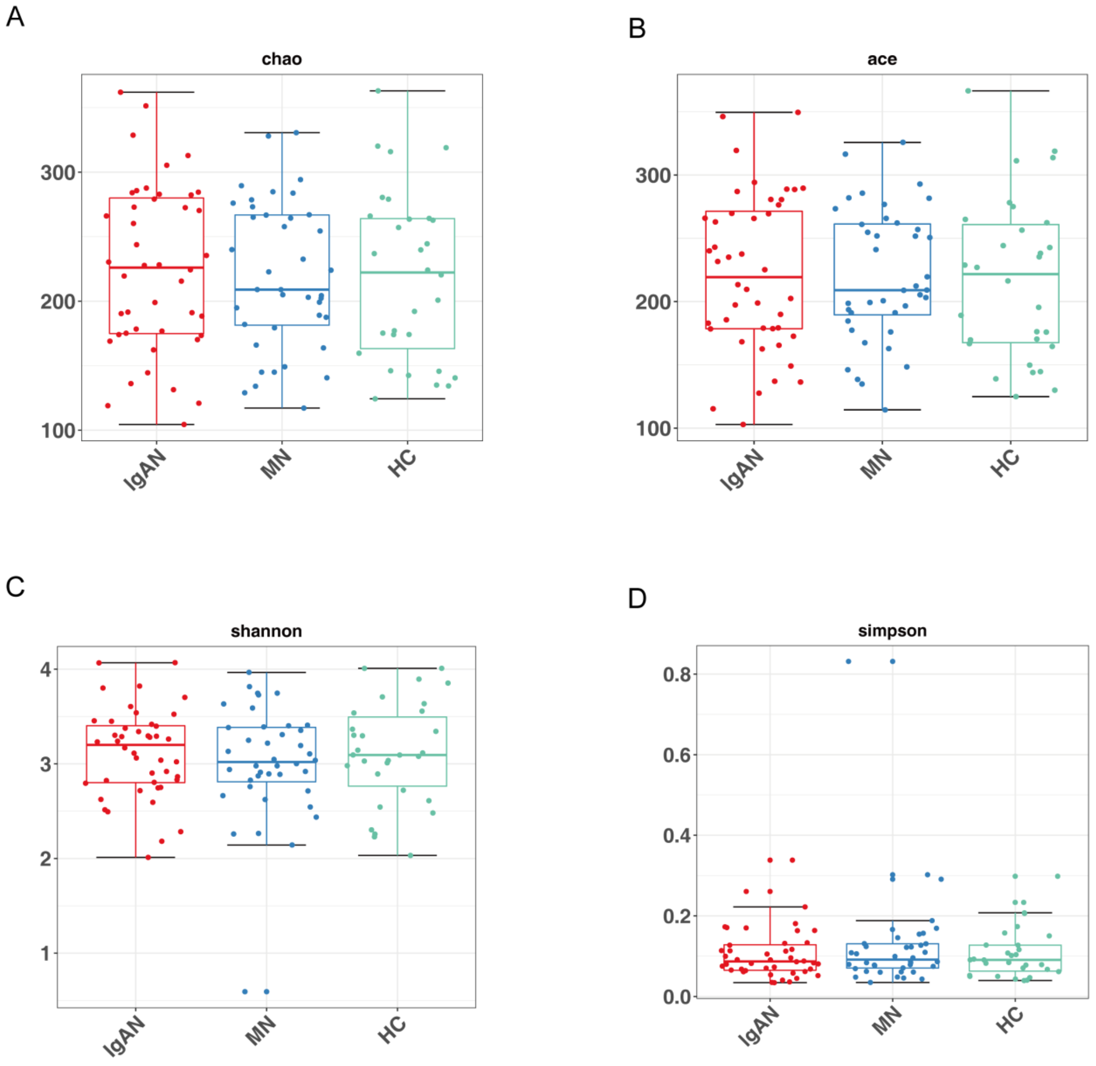

A
chao
300
200
100
IgAN
MN
HC
B
ace
300
200
100
IgAN
MN
HC
C
shannon
4
3
2
1
IgAN
MN
HC
D
simpson
0.8
0.6
0.4
0.2
0.0
IgAN
MN
HC

Supplemental Fig. 1 Community richness and diversity among the three groups. (A) chao indices, (B) ACE indices, (C) Shannon indices and (D) and Simpson indices were similar among the three groups.

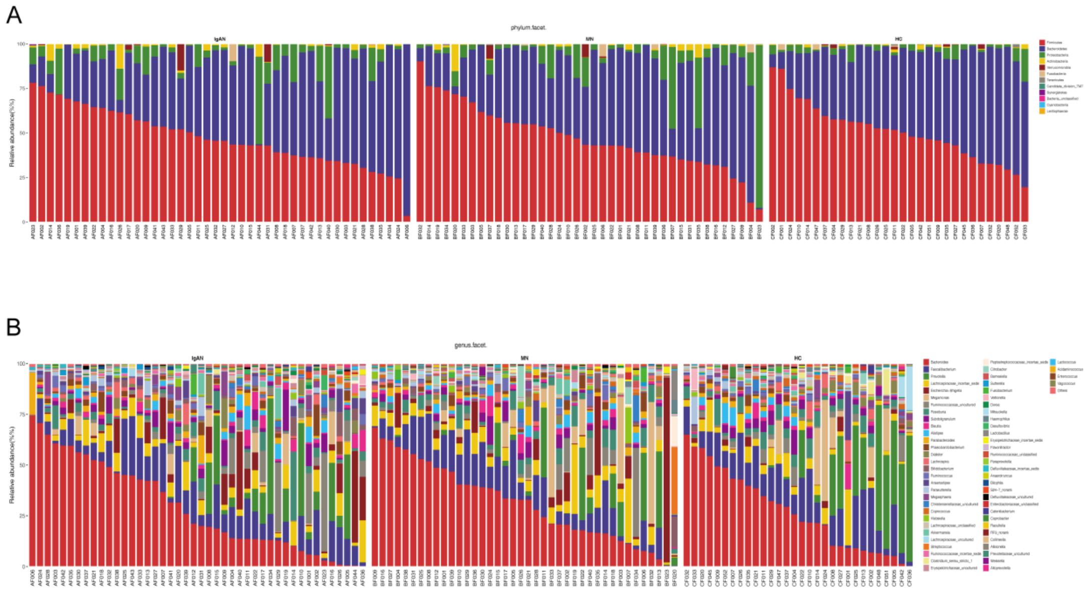

Supplemental Fig. 2 Composition of faecal microbiota among IgAN(n=44), MN(n=40) and HC(n=30). Composition of faecal microbiota among the three groups at the phylum level (A) and the genus level (B). IgAN, immunoglobulin A nedphropathy; MN, membranous nephropathy; HC, healthy controls.

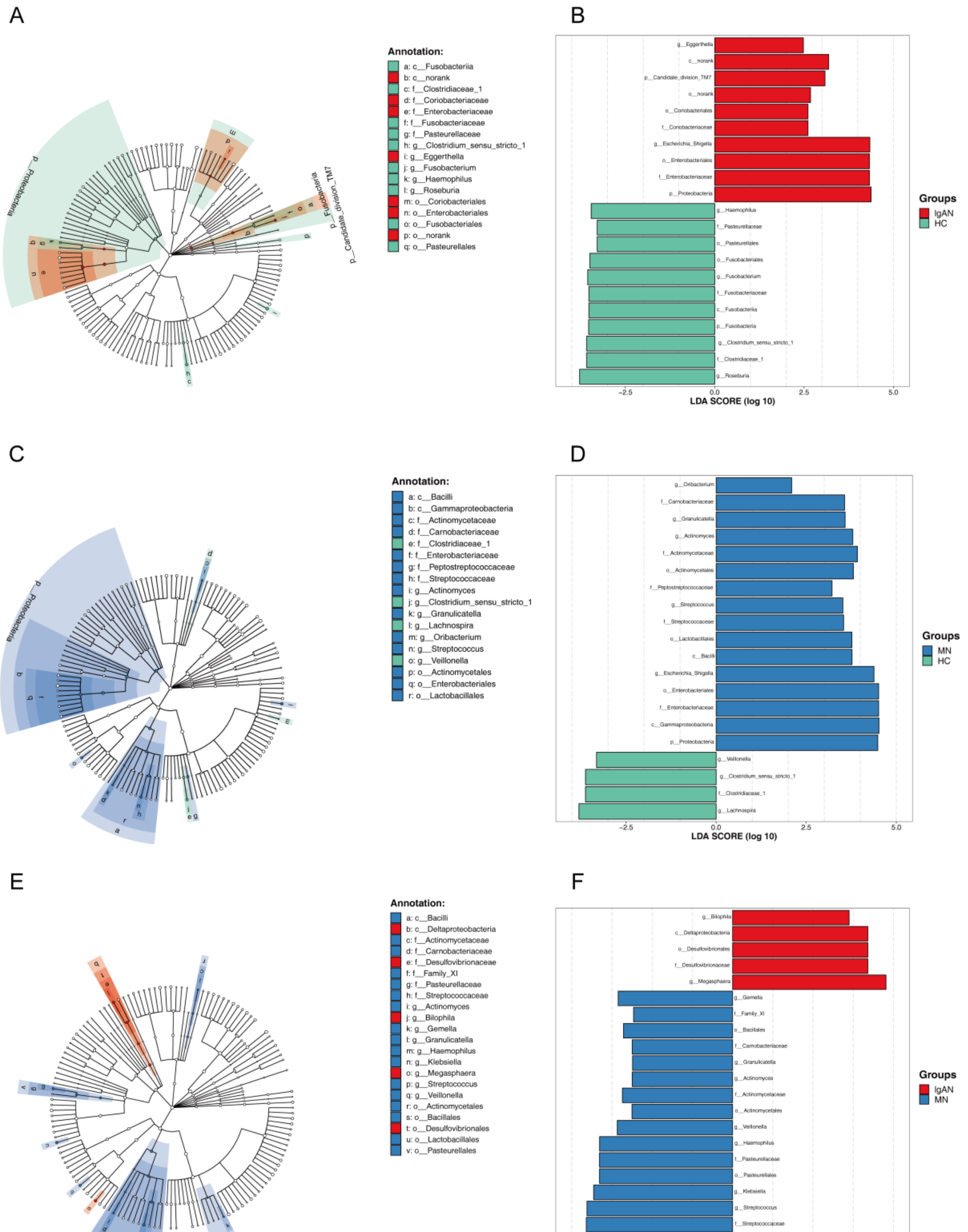
A
B
C
D
E
F
Annotation:
a: c__Fusobacteriia
b: c__norank
c: f__Clostridiaceae_1
d: f__Coriobacteriaceae
e: f__Enterobacteriaceae
f: f__Fusobacteriaceae
g: f__Pasteurellaceae
h: g__Clostridium_sensu_stricto_1
i: g__Eggerthella
j: g__Fusobacterium
k: g__Haemophilus
l: g__Roseburia
m: o__Coriobacteriales
n: o__Enterobacteriales
o: o__Fusobacteriales
p: o__norank
q: o__Pasteurellales
p__Proteobacteria
p__Fusobacteria
p__Candidate_division_TM7
g__Eggerthella
c__norank
p__Candidate_division_TM7
o__norank
o__Coriobacteriales
f__Coriobacteriaceae
g__Escherichia_Shigella
o__Enterobacteriales
f__Enterobacteriaceae
p__Proteobacteria
g__Haemophilus
f__Pasteurellaceae
o__Pasteurellales
o__Fusobacteriales
g__Fusobacterium
f__Fusobacteriaceae
c__Fusobacteriia
p__Fusobacteria
g__Clostridium_sensu_stricto_1
f__Clostridiaceae_1
g__Roseburia
-2.5
0.0
2.5
5.0
LDA SCORE (log 10)
Groups
IgAN
HC
Annotation:
a: c__Bacilli
b: c__Gammaproteobacteria
c: f__Actinomycetaceae
d: f__Carnobacteriaceae
e: f__Clostridiaceae_1
f: f__Enterobacteriaceae
g: f__Peptostreptococcaceae
h: f__Streptococcaceae
i: g__Actinomyces
j: g__Clostridium_sensu_stricto_1
k: g__Granulicatella
l: g__Lachnospira
m: g__Oribacterium
n: g__Streptococcus
o: g__Veillonella
p: o__Actinomycetales
q: o__Enterobacteriales
r: o__Lactobacillales
p__Proteobacteria
g__Oribacterium
f__Carnobacteriaceae
g__Granulicatella
g__Actinomyces
f__Actinomycetaceae
o__Actinomycetales
f__Peptostreptococcaceae
g__Streptococcus
f__Streptococcaceae
o__Lactobacillales
c__Bacilli
g__Escherichia_Shigella
o__Enterobacteriales
f__Enterobacteriaceae
c__Gammaproteobacteria
p__Proteobacteria
g__Veillonella
g__Clostridium_sensu_stricto_1
f__Clostridiaceae_1
g__Lachnospira
-2.5
0.0
2.5
5.0
LDA SCORE (log 10)
Groups
MN
HC
Annotation:
a: c__Bacilli
b: c__Deltaproteobacteria
c: f__Actinomycetaceae
d: f__Carnobacteriaceae
e: f__Desulfovibrionaceae
f: f__Family_XI
g: f__Pasteurellaceae
h: f__Streptococcaceae
i: g__Actinomyces
j: g__Bilophila
k: g__Gemella
l: g__Granulicatella
m: g__Haemophilus
n: g__Klebsiella
o: g__Megasphaera
p: g__Streptococcus
q: g__Veillonella
r: o__Actinomycetales
s: o__Bacillales
t: o__Desulfovibrionales
u: o__Lactobacillales
v: o__Pasteurellales
g__Bilophila
c__Deltaproteobacteria
o__Desulfovibrionales
f__Desulfovibrionaceae
g__Megasphaera
g__Gemella
f__Family_XI
o__Bacillales
f__Carnobacteriaceae
g__Granulicatella
g__Actinomyces
f__Actinomycetaceae
o__Actinomycetales
g__Veillonella
g__Haemophilus
f__Pasteurellaceae
o__Pasteurellales
g__Klebsiella
g__Streptococcus
f__Streptococcaceae
Groups
IgAN
MN

Supplemental Fig. 3 The specific characterization of gut microbiota to identify taxa was analyzed by linear discriminant analysis (LDA) effect size(LEfSe) method between IgAN (n=44) and HC(n=30), between MN(n=40) and HC, between IgAN and MN. (A) LEfSe method identified the most differentially abundant taxons between IgAN and HC. (B) The IgAN-enriched taxa are indicated with a positive LDA score (red), and HC-enriched taxa present a negative score (green). (C) LEfSe method identified the most differentially abundant taxons between MN and HC. (D) The MN-enriched taxa are indicated with a positive LDA score (blue), and HC-enriched taxa present a negative score (green). (E) LEfSe method identified the most differentially abundant taxons between IgAN and MN. (F) The IgAN-enriched taxa are indicated with a positive LDA score (red), and MN-enriched taxa present a negative score (blue). Only taxa achieving an LDA significant threshold >2 are shown.
